# Phase II Trial of MEDI0457 and Durvalumab for Patients With Recurrent/Metastatic Human Papillomavirus-Associated Cancers

**DOI:** 10.1093/oncolo/oyad085

**Published:** 2023-04-27

**Authors:** Van K Morris, Amir Jazaeri, Shannon N Westin, Curtis Pettaway, Solly George, Ryan W Huey, Michaela Grinsfelder, Aaron Shafer, Benny Johnson, David Vining, Ming Guo, Bryan Fellman, Michael Frumovitz

**Affiliations:** Department of Gastrointestinal Medical Oncology, The University of Texas – MD Anderson Cancer Center, Houston, TX, USA; Department of Gynecologic Oncology, The University of Texas – MD Anderson Cancer Center, Houston, TX, USA; Department of Gynecologic Oncology, The University of Texas – MD Anderson Cancer Center, Houston, TX, USA; Department of Urology, The University of Texas – MD Anderson Cancer Center, Houston, TX, USA; Department of Gynecologic Oncology, The University of Texas – MD Anderson Cancer Center, Houston, TX, USA; Department of Gastrointestinal Medical Oncology, The University of Texas – MD Anderson Cancer Center, Houston, TX, USA; Department of Gynecologic Oncology, The University of Texas – MD Anderson Cancer Center, Houston, TX, USA; Department of Gynecologic Oncology, The University of Texas – MD Anderson Cancer Center, Houston, TX, USA; Department of Gastrointestinal Medical Oncology, The University of Texas – MD Anderson Cancer Center, Houston, TX, USA; Department of Radiology, The University of Texas – MD Anderson Cancer Center, Houston, TX, USA; Department of Pathology, The University of Texas – MD Anderson Cancer Center, Houston, TX, USA; Department of Biostatistics, The University of Texas – MD Anderson Cancer Center, Houston, TX, USA; Department of Gynecologic Oncology, The University of Texas – MD Anderson Cancer Center, Houston, TX, USA

**Keywords:** immunotherapy, anti-PD-L1, metastasis, HPV, DNA vaccine

## Abstract

**Background:**

Human papillomavirus (HPV) types 16/18 drive oncogenesis for most patients with cervical, anal, and penile cancers. MEDI0457, a therapeutic DNA vaccine containing plasmids for E6 and E7 HPV-16/18 viral oncogenes and IL-12 adjuvant, is safe and provokes an immune response against E6/E7. We tested MEDI0457 with the anti-PD-L1 antibody durvalumab for patients with HPV-associated cancers.

**Methods:**

Patients with recurrent/metastatic, treatment-refractory HPV-16/18 cervical cancer, or rare HPV-associated (anal and penile) cancers were eligible. Prior immune checkpoint inhibition was not permitted. Patients received MEDI0457 7 mg intramuscularly (weeks 1, 3, 7, 12, and every 8 weeks thereafter) and durvalumab 1500 mg intravenously every 4 weeks. The primary endpoint was overall response (RECIST 1.1). In this Simon two-stage phase 2 trial (H_o_: *p* < 0.15; H_a_: *p* ≥ 0.35), ≥2 responses were needed in both cervical and non-cervical cohorts during the first stage for the trial to proceed to stage 2 with an additional 25 patients (34 total) enrolled.

**Results:**

Twenty-one patients (12 cervical, 7 anal, and 2 penile) were evaluable for toxicity and 19 for response Overall response rate was 21% (95% CI, 6%-46%) among evaluable patients. Disease control rate was 37% (95% CI, 16%-62%). Median duration of response among responders was 21.8 months (95% CI, 9.7%-not estimable). Median progression-free survival was 4.6 months (95% CI, 2.8%-7.2%). Median overall survival was 17.7 months (95% CI, 7.6%-not estimable). Grades 3-4 treatment-related adverse events occurred in 6 (23%) participants.

**Conclusions:**

The combination of MEDI0457 and durvalumab demonstrated acceptable safety and tolerability in patients with advanced HPV-16/18 cancers. The low ORR among patients with cervical cancer led to study discontinuation despite a clinically meaningful disease control rate.

## Teaser

This article evaluates the anti-tumor activity and toxicity profile of the combination of MEDI0457 and durvalumab for patients with recurrent or metastatic HPV-associated anogenital cancers.

**Implications for Practice**

Infection with human papillomavirus drives oncogenesis in the majority of cervical, anal, and penile cancers. Anti-tumor activity with anti-PD-(L)1 antibodies as monotherapy in patients with advanced HPV-associated cancers is modest. This study demonstrated prolonged disease control using combination immunotherapy with a therapeutic HPV-16/HPV-18 DNA vaccine and IL-12 adjuvant in some patients with advanced HPV-associated anogenital malignancies. A minority of patients with HPV-associated cancers experienced durable clinical benefits with durvalumab and MEDI0457 that exceeded one year. Further understanding of immune signatures associated with response to immunotherapy combinations may direct precision approaches of future studies for HPV-associated malignancies.

## Introduction

Infection with oncogenic human papillomavirus (HPV), most commonly types HPV-16 and HPV-18, are responsible for the development of the majority of carcinomas arising from the cervix (>90%),^1,2^ anus (>90%),^3,4^ vagina/vulva (>60%),^5^ and penis (50%).^6^ Development of HPV-associated cancers is mediated by viral oncoproteins E6 and E7, which inhibit tumor suppressor function of p53 and Rb, respectively.^7,8^ In 2022, new diagnoses of squamous cell carcinoma (SCC) of the cervix (>13 000), anal canal (>8500), vagina/vulva (>6000), and penis (>2000) are anticipated.^9^ For some of these HPV-associated cancers (eg, anal cancer), the annual incidence continues to rise in the United States,^10^ a trend expected to continue in the coming decades despite the availability of a preventative vaccine. With rising numbers of new diagnoses of these cancers, therapeutic options which improve survival outcomes must continue to be pursued.

Immune checkpoint blockade therapy anti-programmed death-(ligand)1 (anti-PD-(L)1) antibodies have demonstrated single-agent activity with response rates ranging between 11% and 24% of patients with heavily pretreated, metastatic squamous cell cancers of the cervix, anus, and vagina/vulva.^11-16^ Activity in penile cancer to pembrolizumab has also been noted in case reports.^17^ While the addition of pembrolizumab has demonstrated improved survival when added to cytotoxic chemotherapy specifically for treatment of cervical cancers that express PD-L1,^11^ no other biomarkers predictive for response to immunotherapy are available for use of combination immunotherapy approaches in patients with advanced HPV-associated anogenital malignancies.

MEDI0457 (previously INO-3112; INOVIO Pharmaceuticals) is a plasmid DNA vaccine comprised of 3 plasmids expressing HPV-16 and HPV-18 E6 and E7 proteins and an interleukin-12 (IL-12) plasmid encoding for the adjuvant immune stimulant. In patients with advanced HPV-positive squamous cell cancers of the head/neck and of the cervix, MEDI0457 was safe/well-tolerated and associated with an increased anti-HPV-16/HPV-18 B-cell and T-cell immunity against the E6 and E7 targets.^18,19^ Here, we evaluated the anti-tumor activity and toxicity profile of the combination of MEDI0457 and the anti-PD-L1 antibody durvalumab for patients with recurrent or metastatic HPV-associated anogenital cancers.

## Materials and Methods

### Study Design and Subjects

This is a single-arm, single institution, open-label phase II trial that evaluated the combination of durvalumab and the HPV E6/E7 therapeutic vaccine MEDI0457 for patients with recurrent, metastatic HPV-associated carcinoma. Participants were treated and evaluated in 1 of 2 cohorts based upon the site of their primary tumor: cervical cancer or non-cervical (anal, penile, or vaginal/vulvar) cancer. Confirmation of an HPV-16 or HPV-18 cancer by a Clinical Laboratory Improvement Amendment (CLIA)-certified laboratory was required due to the HPV-16/HPV-18 specificity of MEDI0457.

Participants must have been of 18 years or greater of age; have an advanced cancer refractory to standard therapy; have measurable disease according to Response Evaluation Criteria in Solid Tumors (RECIST) 1.1 criteria; have an Eastern Cooperative Oncology Group performance status of 0 or 1; and have adequate hematologic, renal, and hepatic function. Study participants were not permitted to have received prior corticosteroids or other immunosuppressive medications within 14 days of treatment initiation, nor were they allowed to be taking concurrent therapeutic anticoagulation or irreversible platelet inhibitors (due to repeated intramuscular injections of MEDI0457). Other ineligibility criteria for study participation included a history of primary immunodeficiency, prior antineoplastic immune checkpoint blockade therapy, and active (or prior) autoimmune disease, active infection, and a history of (invasive) second malignancy within 2 years of study entry.

Approval for this clinical trial was granted by the Institutional Review Board at The University of Texas MD Anderson Cancer Center. All patients provided written informed consent prior to study entry. The trial was conducted according to the principles of the Declaration of Helsinki.

### Procedures

Study participants received MEDI0457 at a fixed dose of 7 mg intramuscularly with electroporation using the CELLECTRA device (Inovio) on weeks 1, 3, 7, 12, and every 8 weeks thereafter. Durvalumab was administered at 1500 mg intravenously every 4 weeks beginning at week 4 of study treatment. Radiographic assessments using computerized tomography or magnetic resonance imaging were performed for evaluation of treatment response every 8 weeks. Images of target lesions and metrics for tumor measurements were conducted according to a multimedia structured reporting system called ViSion, which allows for display of serial image findings in graphical disease timelines. The combination of durvalumab and MEDI0457 was continued until disease progression, unacceptable toxicity, withdrawal of informed consent, or death.

For each cohort, dose-limiting toxicities (DLT) were monitored for every 6 patients, with early discontinuation of trial enrollment if Pr(DLT > 30%|data) > 0.90 using a prior beta (0.6, 1.4) for toxicity; that is, given the observed data, if the posterior probability is greater than 0.90 that the true DLT rate is higher than 30%, the trial will be discontinued early. Descriptive statistics were used to summarize toxicities associated with study treatment.

### Outcomes

The primary endpoint for this study was radiographic response according to RECIST version 1.1. Secondary endpoints included adverse events according to Common Terminology Criteria for Adverse Events (CTCAE) version 4.03,^20^ progression-free survival (PFS), overall survival (OS), and disease control at 24 weeks from treatment initiation. The Kaplan-Meier method was performed for estimation of median PFS, OS, and duration of response for each cohort separately and across the entire study population.

### Statistical Analysis

For each of the 2 cohorts of patients (cervical cancer and non-cervical cancer), a Simon two-stage optimal design was utilized. Each cohort was analyzed independently. In the first stage, 9 participants were enrolled. If 0 or 1 response were observed, then treatment with durvalumab and MEDI0457 would be considered futile, and enrollment would be stopped. If there were 2 or more participants per cohort who experienced radiographic responses, an additional 25 patients (34 total) were planned to be enrolled. It was prespecified by study leadership that both cohorts had to surpass the minimum response boundary in the first stage in order for both cohorts to expand into the second stage. Sample sizes were calculated with a one-sided *α* = 0.05 and a *β* = 0.20, using a H_o_: *p* ≤ 0.15 and a H_a_: *p* ≥ 0.35, for which *p* represents the percentage of participants in each cohort who experienced a radiographic response to durvalumab and MEDI0457.

## Results

Between November 2018 and October 2020, there were 21 patients with advanced HPV-associated anogenital cancers treated with durvalumab and MEDI0457: cervical cancer (*N* = 12), anal cancer (*N* = 7), and penile cancer (*N* = 2). There were 4 patients with cervical cancer who presented with adenocarcinomas, whereas all other tumors were of squamous cell histology. The majority (*N* = 18; 95%) of cancers were HPV-16, and there was one HPV-18 (cervical) cancer. As seen in Table 1, the median age for all study participants was 51 years (range, 29-75). Per the Supplementary Table, the mean age of study participants was lower for the cervical cancer cohort than for the non-cervical cancer cohort (42.8 vs. 60.0 years, *P* < .001). The median number of prior therapies was 2 (range, 1-4). Among those with anal cancer, there were 6 female and 1 male participants. Using available archival tissue when available, a PD-L1 + status was detected in 5 of 9 (56%) samples. Among the ­non-cervical cancer cohort, only one anal cancer was tested for PD-L1 expression, as routine testing for this biomarker is not performed due to the lack of predictive utility for immunotherapy reported thus far.

**Table 1.** Characteristics of study participants evaluable for response (*N* = 19).

Characteristic	*N*	%
Age		
Mean, years (range)	51.0 (29-75)	
Gender		
Female	16	84.2
Male	3	15.8
Cohort		
Cervical	10	52.6
Rare-anal	7	36.8
Rare-penile	2	10.5
Histology		
Adenocarcinoma	4	21.1
squamous	15	78.9
HPV status		
HPV 16+	18	94.7
HPV 18+	1	5.3

**Figure 1.**
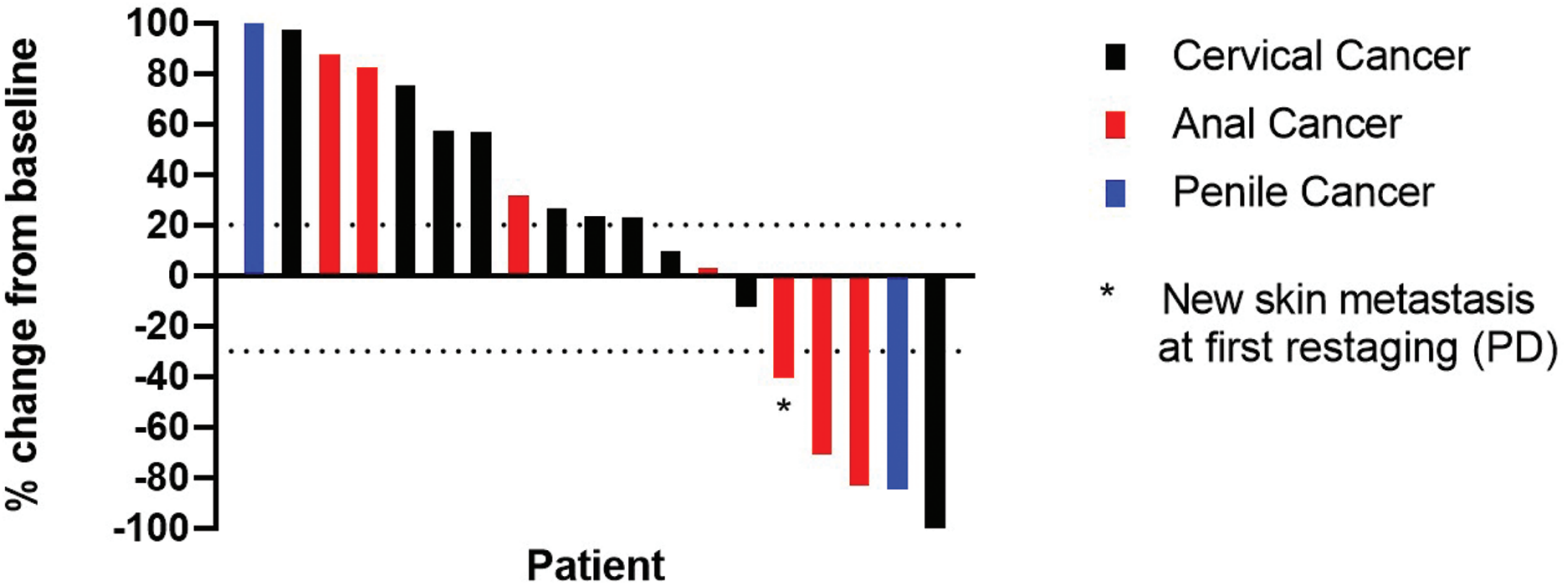
Waterfall plot.

There were 19 patients evaluable for the primary endpoint—10 in the cervical cancer cohort and 9 in the ­non-cervical cancer cohort. Two patients were not evaluable for response due to rapid clinical deterioration. There was 1 participant with cervical cancer who experienced a complete radiographic response, and 3 participants (2 with anal cancer and 1 with penile cancer) with partial responses. For the entire study population (Fig. 1), the overall response rate (ORR) was 21.1% (95% CI, 6.1%-45.6%) among evaluable patients and 19% (4/21, 95% CI, 5%-42%) in the ­per-protocol population. For evaluable patients, the median duration of response was 21.8 months (95% CI, 9.7%-not estimable). The disease control rate (DCR) for durvalumab and MEDI0457 was 36.8% (95% CI, 16.3%-61.6%). Table 2 lists the best radiographic responses according to the individual cohorts. For evaluable participants with cervical cancer, the ORR was 10.0% (95% CI, 0.3%-44.5%), and the DCR was 30.0% (95% CI, 6.7%-65.2%). The ORR in the non-cervical cancer cohort was 33.3% (95% CI, 7.5%-70.1%), and the DCR was 44.4% (95% CI, 13.7%-78.8%). Because the ORR for the cervical cancer cohort did not satisfy the criteria for expansion into the second stage per the study design, the study team made the decision not to proceed with further enrollment for either cohort.

**Table 2.** Best radiographic response.

	Cervical		Non-cervical	
Characteristic	*N*	%	*N*	%
Best overall response				
PD	7	70.0	4	44.4
SD	2	20.0	2	22.2
PR	0	0.0	3	33.3
CR	1	10.0	0	0.0

**Figure 2.**
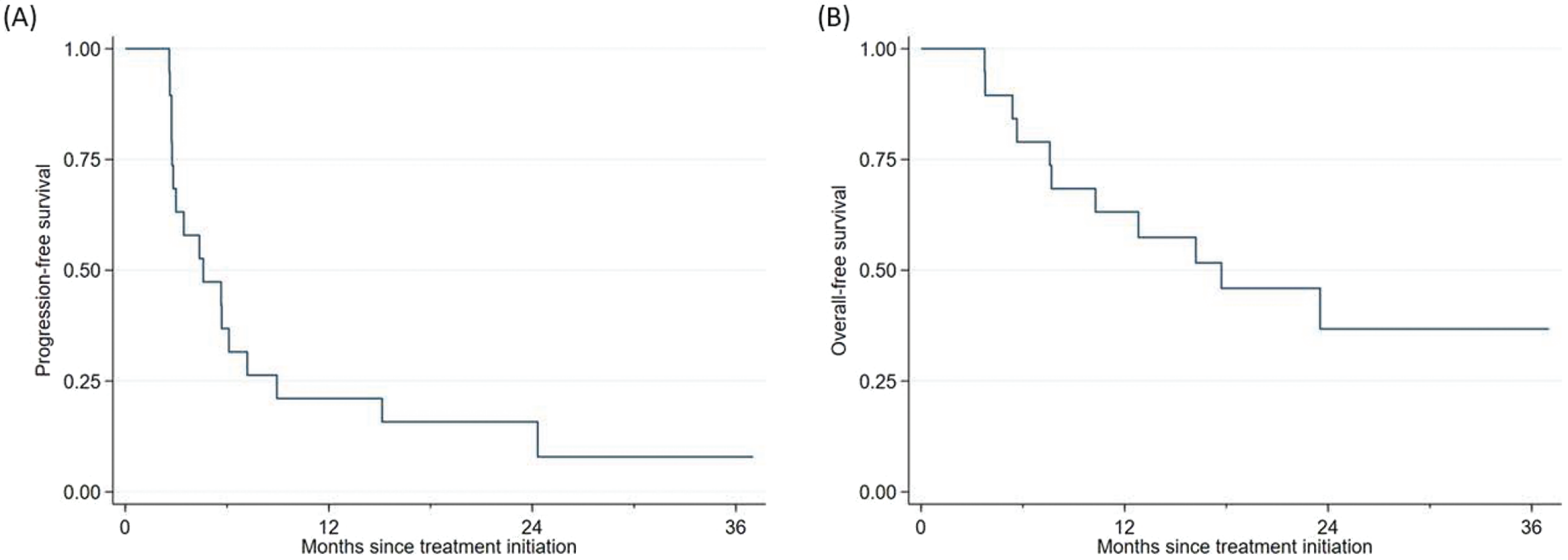
Progression-free survival (**A**); overall survival (**B**).

As seen in Fig. 2A, the median PFS for durvalumab and MEDI0457 among all evaluable patients was 4.6 months (95% CI, 2.8%-7.2%). Median PFS outcomes were similar between the cervical cancer (4.6 months; 95% CI, 2.6%-6.1%) and non-cervical cancer (4.4 months; 95% CI, 2.6%-not estimable) cohorts. Rates of 1-year PFS for participants with advanced cervical and non-cervical HPV-associated cancers were estimated to be 10.0% (95% CI, 0.6%-35.8%) and 33.3% (95% CI, 7.8%-62.3%), respectively.

Median OS (Fig. 2B) for durvalumab and MEDI0457 across the entire study population was 17.7 months (95% CI, 7.6%-not estimable). For participants with cervical cancer, the median OS was 7.6 months (95% CI, 3.7%-17.7%). Separately, median OS was not estimable in the non-cervical cancer cohort.

Overall, durvalumab and MEDI0457 demonstrated acceptable safety and tolerability (Table 3), with adverse events for the combination consistent with prior reports of these agents individually. No grade 5 treatment-related adverse events occurred. Grade ≥ 3 treatment-related adverse events included a grade 4 (asymptomatic) elevated serum lipase level (*N* = 1), and grade 3 arthritis, cholecystitis, ascites, elevated liver function tests, hypokalemia, hyponatremia, neutropenia, and asymptomatic elevated serum lipase level (all *N* = 1). Only one patient discontinued study treatment due to a ­pembrolizumab-related inflammatory arthritis. For this patient (with penile cancer), his symptoms resolved following corticosteroids, and he remains without evidence of disease progression while off any subsequent therapy. Across all grades, adverse events related to durvalumab and MEDI0457 which occurred in >20% of participants included localized reaction at the MEDI0457 injection site (*N* = 21; 100%), fatigue (*N* = 10; 48%), extremity pain at the site of MEDI0457 injection (*N* = 5; 24%), and increased liver function tests (*N* = 5; 23%).

**Table 3.** Treatment-related adverse events.

Adverse event	Maximum grade				
	1	2	3	4	Total
Abdominal pain	3	0	0	0	3
Alanine aminotransferase—increased	4	0	1	0	5
Alkaline phosphatase—increased	2	0	1	0	3
Anorexia	1	0	0	0	1
Anxiety	1	1	0	0	2
Arthralgia	1	0	0	0	1
Arthritis	0	0	1	0	1
Ascites	0	0	1	0	1
Aspartate aminotransferase—increased	3	0	1	0	4
Blood bilirubin increased	2	0	0	0	2
CPK increased	2	0	0	0	2
Cholecystitis	0	0	1	0	1
Cough	1	0	0	0	1
Creatinine increased	0	1	0	0	1
Dizziness	1	0	0	0	1
Dry mouth	0	1	0	0	1
Dyspnea	0	1	0	0	1
Eosinophilia	1	0	0	0	1
Fatigue	7	3	0	0	10
Fever	1	0	0	0	1
Headache	1	0	0	0	1
Hyperglycemia	1	0	0	0	1
Hyperthyroidism	1	1	0	0	2
Hypokalemia	1	1	1	0	3
Hyponatremia	1	0	1	0	2
Hypothyroidism	0	4	0	0	4
Injection site reaction	0	21	0	0	21
Lipase increased	2	1	0	1	4
Lymphopenia	3	0	0	0	3
Mucositis oral	0	1	0	0	1
Nausea	3	1	0	0	4
Neutropenia	0	0	1	0	1
Pain	0	1	0	0	1
Pain in extremity	1	4	0	0	5
Thrombocytopenia	2	0	0	0	2
Rash maculo-papular	3	0	0	0	3
Serum amylase increased	2	0	1	0	3
Sinus bradycardia	1	0	0	0	1
Vomiting	1	0	0	0	1

## Discussion

While the benefit of combination immunotherapy has yet to be demonstrated in patients with previously treated, advanced HPV-associated anogenital cancers, we present here intriguing pilot data that demonstrates prolonged disease control in multiple participants following treatment with durvalumab and the HPV-16/18 E6/E7 DNA vaccine MEDI0457. Here, durable responses occurred in persons with cervical cancer, anal cancer, and penile cancer alike. Overall, this treatment combination appeared safe and well-tolerated, with no DLTs observed and no suggestion of unacceptable toxicity.

Anti-PD-1 antibodies as monotherapy have yielded modest response rates for treatment of incurable HPV-positive cancers. For example, in patients with advanced cervical and anal cancers, pembrolizumab demonstrated response rates of 12% and 10%, respectively.^11,13^ Other single-arm studies in this setting have reported similar response rates.^12,14,21^ Combinations of immunotherapy with selected targeted therapies have not suggested further improvement thus far. For example, atezolizumab and the anti-vascular endothelial growth factor antibody bevacizumab demonstrated an overall response rate of 10% in patients with advanced anal cancer,^15^ and avelumab with the anti-epidermal growth factor antibody cetuximab revealed an overall response rate of 17% in a similar population.^14^ In contrast, promising early signal has been seen in other trials for patients with advanced HPV-associated cancers using anti-PD-L1 therapies in combination with therapeutic HPV vaccines. For example, a phase II trial of PDS0101 (HPV-16 E6/E7 & peptide vaccine), M9241 (Il-12 targeting immunocytokine), and bintrafusp alfa (anti-PD-L1/TGF-β bifunctional protein) demonstrated an overall response rate of 43%.^22^ Therefore, novel therapeutic approaches that increase immune-mediated anti-tumor activity with immune checkpoint blockade agents, especially with therapeutic HPV vaccines, are warranted.

In our study, the overall response rate of durvalumab and MEDI0457 demonstrated an ORR of 21%. Notably, the cervical cancer and non-cervical cohorts were conducted independently in parallel, and responses were observed in 10% and 33% of participants, respectively. While the response rate in the initial stage did surpass the prespecified boundary for expansion in the non-cervical (anal/penile) cancer group, these criteria were not satisfied in the cohort of patients with cervical cancer, with a response noted only in 1 patient. Because the required response rate was not satisfied in all cohorts, further expansion for additional patients with advanced HPV-associated cancers to receive durvalumab and MEDI0457 was not pursued.

Median progression-free survival for the entire study population was 4.6 months and similar for both cohorts. Those participants were able to experience durable disease control suggests that some patients with HPV-associated malignancies may derive clinical benefits with stimulation by a therapeutic vaccine targeting HPV-specific antigens. In a separate study of patients with advanced HPV-16 head/neck, cervical, and anal cancers, treatment with nivolumab and an HPV-16 specific synthetic peptide achieved an overall response rate of 33% and generated an increase in HPV-16 T cells within the tumor microenvironment.^23^ Further understanding of characteristics of patients with HPV-associated cancers who may benefit from immune priming by such therapeutic vaccines like MEDI0457 are important to build upon future immunotherapy trial designs.

We recognize limitations associated with generalizing our trial findings. Many patients received 3 or more lines of prior systemic therapy, which has been shown in other cancers to promote an immune-suppressed tumor microenvironment that may blunt responses to immunotherapy. Nonetheless, we are encouraged that some participants in our study were able to achieve extended clinical benefits with the combination of durvalumab and MEDI0457 beyond what has historically been reported with anti-PD-(L)1 antibodies as monotherapy in patients with advanced HPV-associated cancers. Based on our findings, we support continued investigation of novel strategies in combination with immune checkpoint blockade that prime the tumor microenvironment for rationally designed immune-mediated attack.

## Summary/Conclusions

We demonstrate prolonged disease control using combination immunotherapy with a therapeutic HPV-16/HPV-18 DNA vaccine and IL-12 adjuvant in some patients with advanced HPV-associated anogenital malignancies. A minority of patients with HPV-associated cancers experienced durable clinical benefit with durvalumab and MEDI0457 that exceeded 1 year.

## Supplementary Material

oyad085_suppl_Supplementary_Table

## Data Availability

The data underlying this article are available in the article and in its online supplementary material.
