# Supplementary material for: Phase II Trial of MEDI0457 and Durvalumab for Patients With Recurrent/Metastatic Human Papillomavirus-Associated Cancers
Source: Oncologist. 2023 Apr 27;28(7):618–23. doi: 10.1093/oncolo/oyad085 (PMC10322132; doi:10.1093/oncolo/oyad085)
Supplement: oyad085_suppl_Supplementary_Table [file oyad085_suppl_supplementary_table.docx]

|  | **Cervical** | | **Non-cervical** | |
| --- | --- | --- | --- | --- |
| **Characteristic** | **N** | **%** | **N** | **%** |
| Age |  |  |  |  |
| N | 10 | | 9 | |
| Mean (Standard dev) | 42.8 (8.3) | | 60.0 (9.5) | |
| Gender |  |  |  |  |
| F | 10 | 100.0 | 6 | 66.7 |
| M | 0 | 0.0 | 3 | 33.3 |
| Histology |  |  |  |  |
| Adenocarcinoma | 4 | 40.0 | 0 | 0.0 |
| squamous | 6 | 60.0 | 9 | 100.0 |
| HPV status |  |  |  |  |
| HPV 16+ | 9 | 90.0 | 9 | 100.0 |
| HPV 18+ | 1 | 10.0 | 0 | 0.0 |

**Supplemental Table: Demographic and clinical characteristics by cohort**
